# A pothole video dataset for semantic segmentation

**DOI:** 10.1016/j.dib.2024.110131

**Published:** 2024-02-01

**Authors:** Muhammad Ihsan, Muhammad Alfian Amrizal, Agus Harjoko

**Affiliations:** Department of Computer Science and Electronics, Universitas Gadjah Mada, Indonesia

**Keywords:** Artificial intelligence, Computer vision, Deep learning, Road damage, Road potholes, Segmentation, Sequence data

## Abstract

This paper introduces a video dataset for semantic segmentation of road potholes. This dataset contains 619 high-resolution videos captured in January 2023, covering locations in eight villages within the Hulu Sungai Tengah regency of South Kalimantan, Indonesia. The dataset is divided into three main folders, namely train, val, and test. The train, val, and test folders contain 372 videos for training, 124 videos for validation, and 123 videos for testing, respectively. Each of these main folders has two subfolders, ``RGB'' for the video in the RGB format and ``mask'' for the ground truth segmentation. These videos are precisely two seconds long, containing 48 frames each, and all are in MP4 format. The dataset offers remarkable flexibility, accommodating various research needs, from full-video segmentation to frame extraction. It enables researchers to create ground truth annotations and change the combination of videos in the folders according to their needs. This resource is an asset for researchers, engineers, policymakers, and anyone interested in advancing algorithms for pothole detection and analysis. This dataset allows for benchmarking semantic segmentation algorithms, conducting comparative studies on pothole detection methods, and exploring innovative approaches, offering valuable contributions to the computer vision community.

Specifications Table

**Table:** 

Subject	Computer Vision and Pattern Recognition
Specific subject area	Semantic Segmentation for road potholes
Data format	Raw and segmentation videos
Type of data	.mp4
Data collection	Data was captured utilizing the Xiaomi Mi 10T smartphone, initially recording in 4 K resolution. The video was shot top-down to the road, maintaining a tilt angle of approximately 80–100° and around 130 cm between the camera and the pothole. Subsequently, the data was subjected to cropping and resizing using Adobe Premiere Pro, resulting in a resolution of 1080 × 1080 and an aspect ratio of 1:1. After that, the pen tool and mask tracker tools in Adobe After Effects were used to create the ground truth segmentation.
Data source location	City: Hulu Sungai Tengah Country: Indonesia Latitude and Longitude: −2.6153°N, 115.5207°E
Data accessibility	Repository name: Mendeley Data [1] Data identification number: 10.17632/5bwfg4v4cd.2 Direct URL to data: https://data.mendeley.com/datasets/5bwfg4v4cd/2

## 1 Value of the Data

• This dataset offers a new feature which is moving pothole data recorded by a camera. Currently available pothole data comprises solely of independent and still images [2], [3], [4], [5], [6]. The introduction of this continuous video annotation data will unlock opportunities for advancing and refining novel techniques in pothole detection and segmentation.
• This dataset presents a semi-manually annotated ground truth label of each pixel in the video frames that represents the shape and size of the potholes. Such data is crucial for the development of better pothole detection and segmentation models that are aimed not only to detect whether there is a pothole or not, but also to show the precise shape and size of the pothole.
• In real-world application, this dataset holds significance for researchers in computer vision and deep learning, providing an opportunity to experiment, develop, and create an intelligent system that can be used for automatic pothole size estimation, facilitating road damage assessment using vehicles equipped with cameras.

## 2 Background

To ensure the safety of road users, routine inspection and repair of potholed roads are crucial tasks to be carried out by the authorities. However, manual inspection is very time-consuming and cost inefficient, especially in developing countries where potholes are very common. Therefore, many researchers have proposed and developed systems to detect potholes more efficiently using digital image processing techniques. These systems usually comprise an embedded system equipped with camera and attached to a car. The camera records the road condition, and an artificial intelligence (AI), trained with pothole images, is used to determine the level of the road damage. However, we found out that currently, researchers have only trained their AI models using independent and still images, for example, with pothole image dataset such as [2]. The resulting AI might be not optimal because the model input data captured by the camera consists of continuous frames, where the position of a pothole in one frame is related to the subsequent frames. Exploiting the correlation between frames will allow the development of real-time road damage prediction system and hence, a new dataset for supporting this development is needed.

## 3 Data Description

**Fig. 1:**
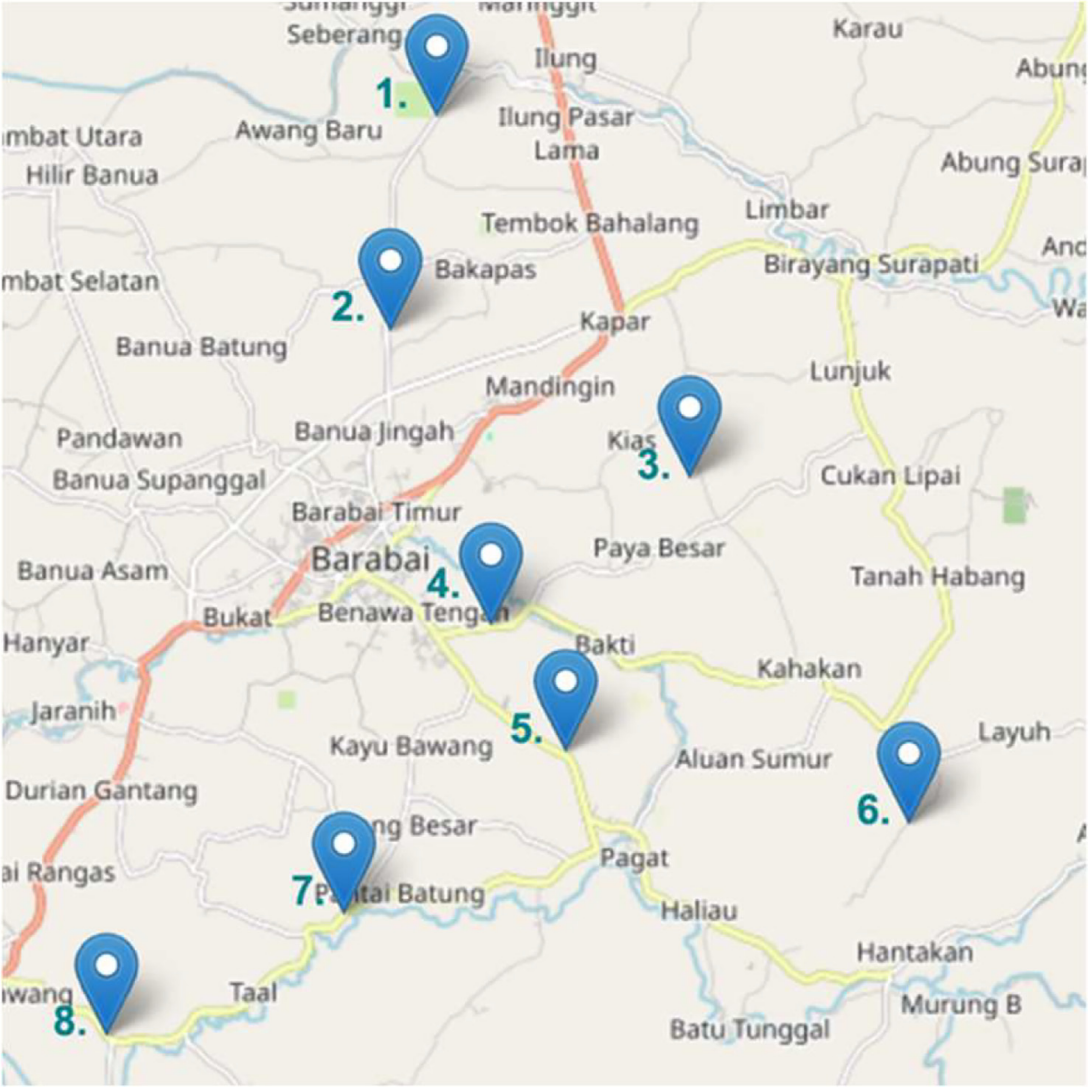
Detailed locations of the data collection.

This dataset contains 619 video recordings of road potholes, all captured between January - February 2023. We filmed these videos in various locations spanning eight villages in the Hulu Sungai Tengah regency of South Kalimantan, Indonesia. Fig. 1 and Table 1 show the more specific shooting locations.

**Table 1:** Brief description about the data collection.

No	Particulars	Description
1.	Road Damage	Pothole
2.	Shooting Period	30 January - 03 February 2023
3.	Detailed Geographical Location	(1) Ayuang: −2.5272°N, 115.3933°E (2) Banua Jingah: −2.5554°N, 115.3869°E (3) Paya: −2.5752°N, 115.4267°E (4) Aluan: −2.5948°N, 115.4003°E (5) Gambah: −2.6116°N, 115.4102°E (6) Haliau: −2.6212°N, 115.4559°E (7) Murung Taal: −2.6332°N, 115.3809°E (8) Banua Kepayang: −2.6491°N, 115.3492°E
4.	Climate	Sunny and Cloudy
5.	Original Video Size	2160 × 3840
6.	Total Videos	619

**Fig. 2:**
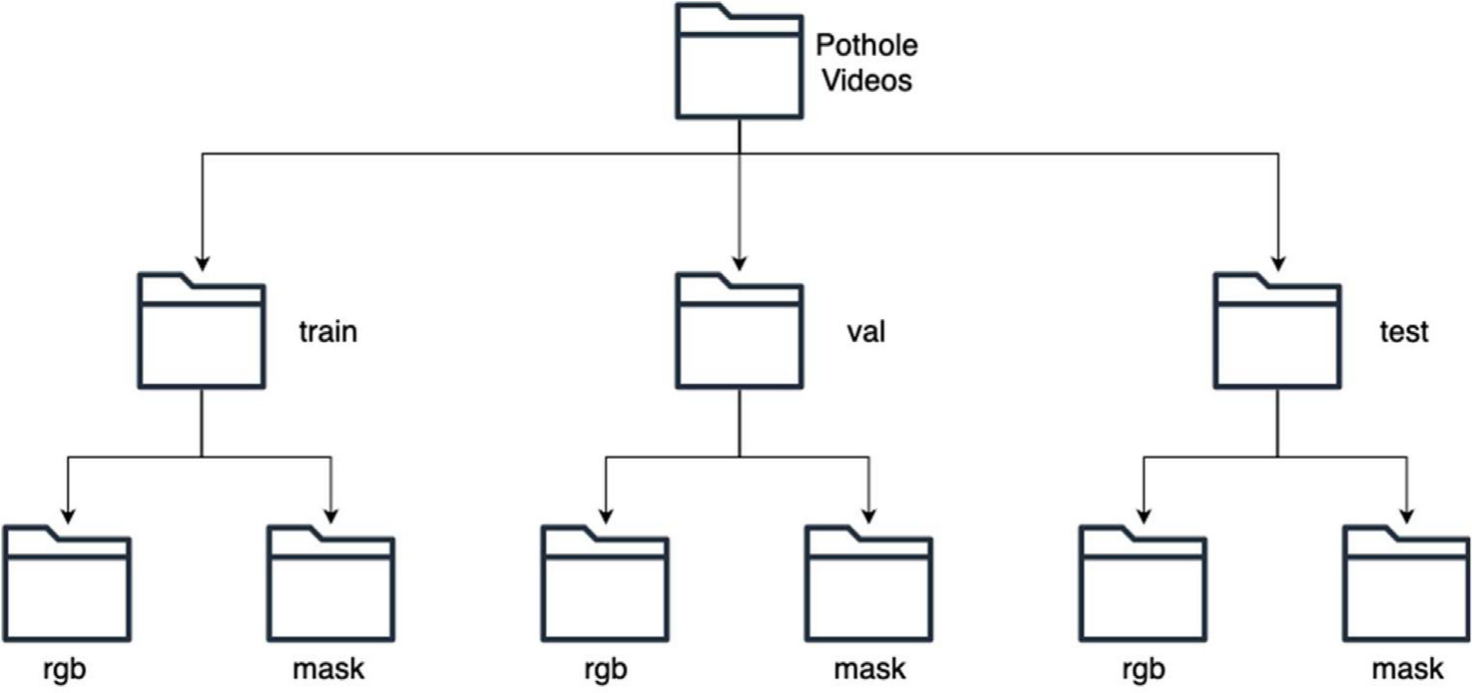
Folder structure in the dataset.

We categorized the collected data into three main folders: train, test, and val. The train folder consists of 372 videos for training, the val folder consists of 124 videos for validation, and the test folder consists of 123 videos for testing. In each folder, we divided the data into two additional subfolders named rgb and mask. The rgb folder contains the original video, while the mask folder contains the annotated videos showing the ground truth of each pixel, either pothole or non-pothole. Fig. 2 illustrates the hierarchical structure of the dataset hosted on Mendeley Data.

**Fig. 3:**
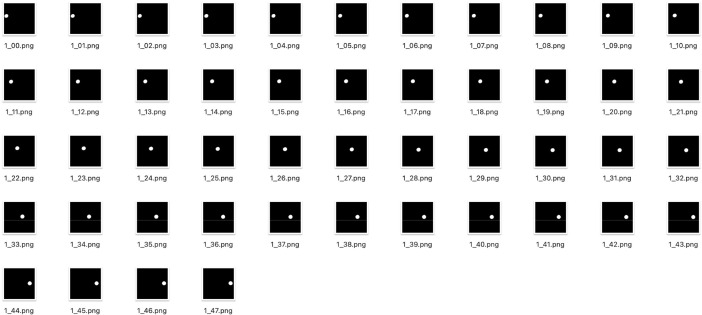
Example frames of a ground truth video.

The original RGB and ground truth videos uniformly have a duration of two seconds, each comprising 48 frames. Fig. 3 displays example frames of a ground truth video of this dataset. The white and black pixels represent pothole and non-pothole, respectively. Both the RGB and ground truth videos are delivered in high-resolution MP4 format. Therefore, future researchers can use this dataset with a lot of flexibility; it enables them to use it in various scenarios, such as extracting individual frames from the video, creating custom ground truth annotations, or establishing personalized training, testing, and validation data.

## 4 Experimental Design, Materials and Methods

In this section, we discuss the data collection methods, pre-processing, and the creation of ground truth segmentation. In each subsection, a concise explanation of the techniques and tools used, along with their illustrations, are provided.

### 4.1 Data collection

**Fig. 4:**
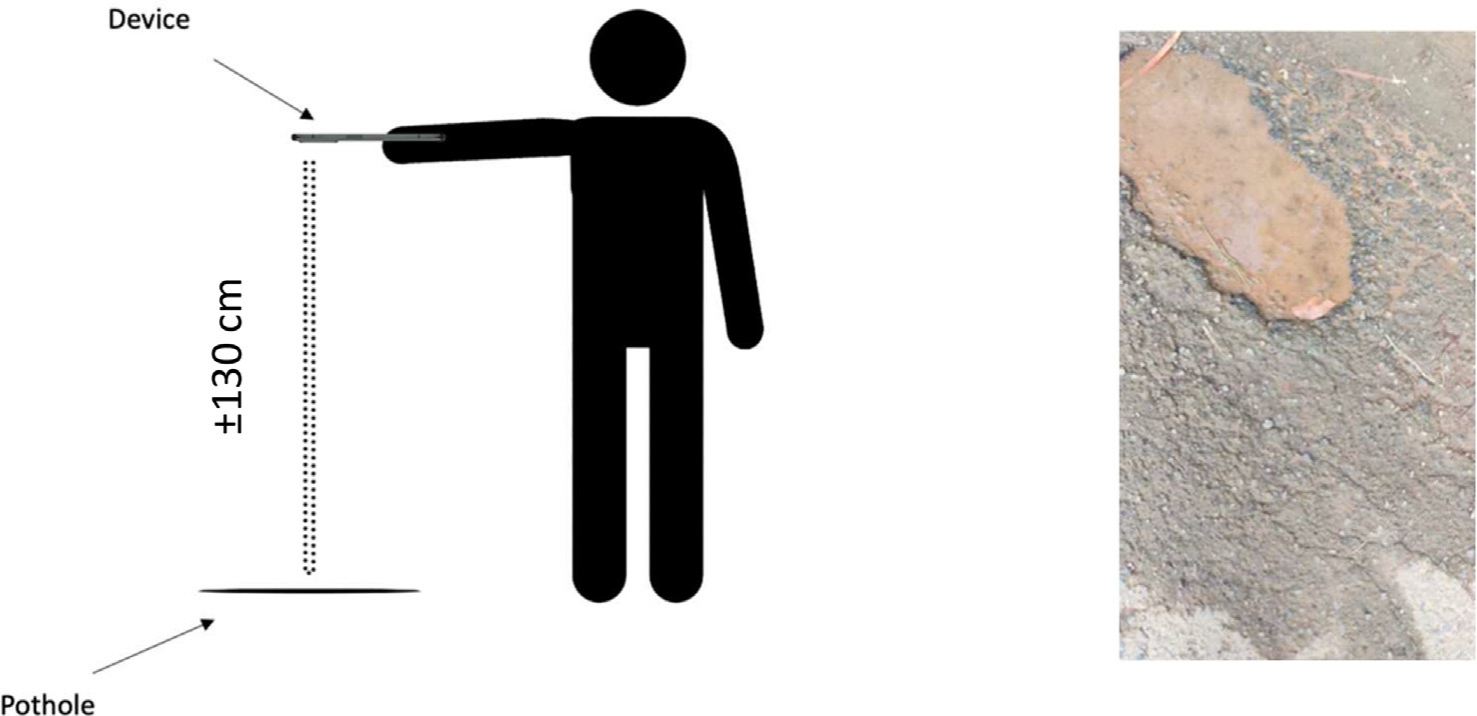
Illustration of data acquisition (left), sample obtained results (right).

The data was collected using a Xiaomi Mi10T cell phone camera with a resolution of 2160 × 3840. Our data collection approach was inspired by the Pothole 600 dataset [2], resulting in top-down-oriented videos capturing road potholes directly. The distance between the camera and the pothole varied around 130 cm (Fig. 4) depending on terrain and camera placement. When taking a video, the pothole is intentionally positioned in the top half of the camera screen to make the pre-processing stage easier. We gathered a total of 619 video clips, each originally in the duration of 2–3 s.

The purpose of capturing the video in a top-down manner is because this dataset is originally intended for training a deep learning model that can automatically determine the size of road potholes on vehicles equipped with top-down-directed cameras, such as in [6]. Positioning the camera in this way will make it easier to measure the area of road potholes automatically compared to placing the camera on the car dashboard as in [7].

### 4.2 Data pre-processing

**Fig. 5:**
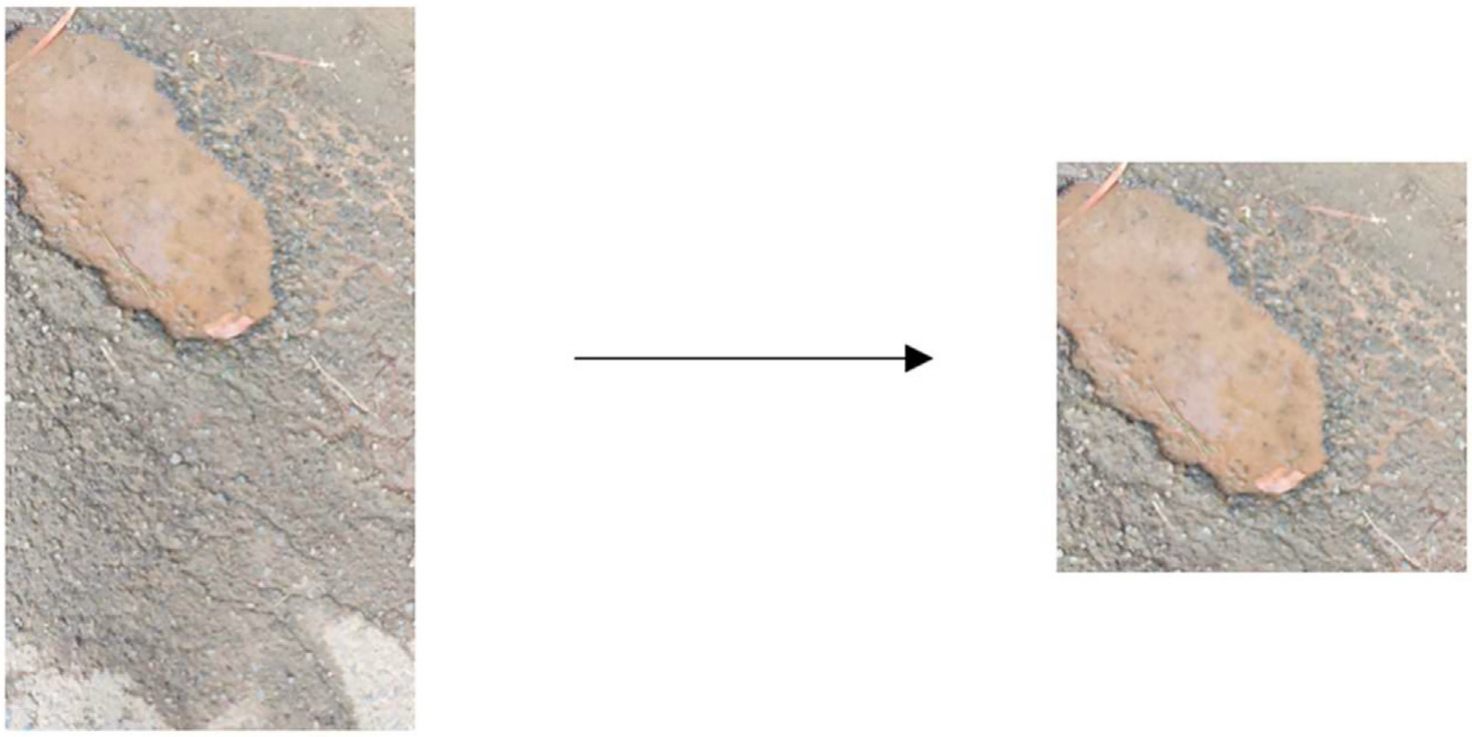
The resulting video frame after cropping and resizing.

Before labeling the ground truth, we adjust all acquired data through cropping and resizing using Adobe Premiere Pro application [8]. We cropped the bottom half image in each frame of the video so that the resulting video specifically focused on the road pothole area. Then, resizing is performed aiming to reduce the size of the resulting file, so that the next step, ground truth generation stage, can be more efficient. After cropping and resizing, the outputs are in the form of RGB videos with a 1:1 aspect ratio and 1080 × 1080 pixels. Other than cropping and resizing, video durations were standardized to two seconds of duration at a frame rate of 24 fps, ensuring each video contains 48 image frames. Fig. 5 shows an example of a video frame before and after the preprocessing stage.

### 4.3 Creation of ground truth segmentation data

**Fig. 6:**
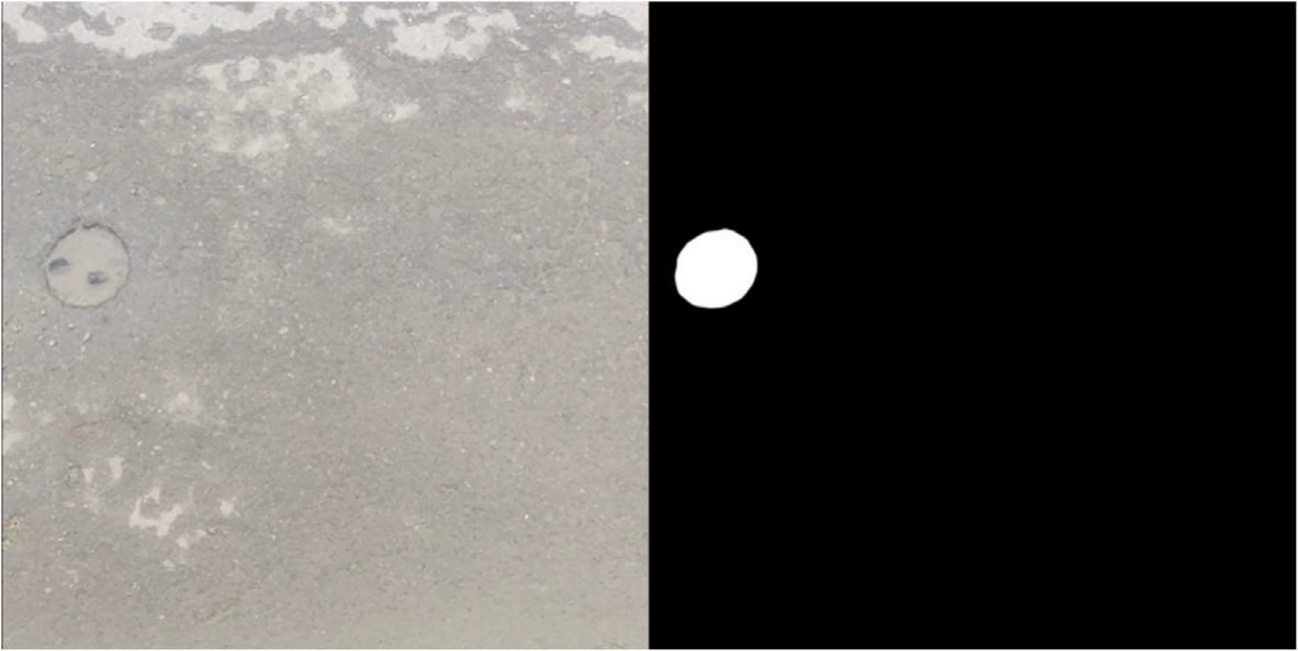
RGB frame (left), ground truth segmentation (right).

Next, we semi-manually annotated the pre-processed videos using Adobe After Effects application [9]. The original RGB videos were transformed into black-and-white videos representing the segmentation results. The pen tool in the After Effects is used for the annotation. We performed manual annotation on a single image frame, and After Effects automatically generated the remaining annotated frames using the mask-tracking feature, hence we called it “semi-manual.” The final step is checking the masked results on each frame, ensuring the quality of the final segmented masked video. Fig. 6 shows an example of a video frame and its ground truth segmentation.

## Limitations

In certain videos, the boundary between potholes and non-potholes areas are not clear. This makes the manual annotation using the pen tool might not be 100% accurate. When the manual annotation is less accurate, this annotation result will propagate to other frames in the video because the remaining frames were automatically annotated using After Effects's mask tracking feature. Consequently, the ground truth outcomes may not be as perfect as when a camera with depth perception is used, as in the Pothole-600 dataset [2].

## Ethics Statement

We confirm that we have thoroughly reviewed and adhered to the ethical guidelines for publication in Data in Brief. The research presented in this work does not involve human subjects, animal experiments, or the utilization of data collected from social media platforms.

## CRediT authorship contribution statement

**Muhammad Ihsan:** Data curation, Investigation, Visualization, Writing – original draft. **Muhammad Alfian Amrizal:** Conceptualization, Project administration, Writing – review & editing, Validation, Supervision. **Agus Harjoko:** Methodology, Validation, Supervision.

## Data Availability

Pothole Videos (Original data) (Mendeley Data).
